# Cholesterol as a Risk Factor for Subarachnoid Hemorrhage: A Systematic Review

**DOI:** 10.1371/journal.pone.0152568

**Published:** 2016-04-14

**Authors:** Joni Valdemar Lindbohm, Jaakko Kaprio, Miikka Korja

**Affiliations:** 1 Department of Public Health, University of Helsinki, P.O. Box 41, FI-00014, Helsinki, Finland; 2 Department of Neurosurgery, University of Helsinki and Helsinki University Hospital, P.O. Box 266, FI-00029, Helsinki, Finland; 3 National Institute for Health and Welfare, P.O. Box 30, FI-00271, Helsinki, Finland; 4 Institute for Molecular Medicine FIMM, P.O. Box 20, FI-00014, Helsinki, Finland; University of Glasgow, UNITED KINGDOM

## Abstract

**Background:**

The role played by total cholesterol (TC) in risk for subarachnoid hemorrhage (SAH) is unclear because studies report both high and low TC each as a risk factor. We performed a systematic review to clarify associations between lipid profile and SAH.

**Methods:**

Our literature search comprised Pubmed, Scopus, and Cochrane Library databases with no language, publication year, or study type limitations. The Preferred Reporting Items for Systematic reviews and Meta-analyses (PRISMA) checklist guided our reporting. Data forms adapted from the Critical Appraisal Skills Program (CASP), and Cochrane Collaboration guidelines provided a platform for risk-of-bias evaluation. We used a random effects model to calculate pooled estimates and assessed heterogeneity with I^2^-statistics.

**Results:**

Of the final 21 studies reviewed, 12 were prospective and 9 retrospective. All studies assessed TC, four assessed HDL, and none LDL in risk for SAH. Heterogeneity among all, retrospective, and Asian studies was high (I^2^ = 79.5%, I^2^ = 89.0%, and I^2^ = 84.3%) and considerable in prospective (I^2^ = 46.0%). We therefore focused on qualitative analysis and found that only two studies had a low risk of bias. According to these studies high TC increases risk for SAH in men, whereas the role of HDL remained unclear.

**Conclusion:**

The low-risk-of-bias studies suggest that elevated TC levels elevate risk for SAH in men. Due to the high prevalence of hypercholesterolemia, population attributable risk (PAR) of hypercholesterolemia may exceed the PARs of smoking and hypertension in men. Apart from diabetes and obesity, the risk-factor profile of SAH seems to resemble that of other cerebrovascular diseases, at least in men.

## Introduction

Epidemiological studies of risk factors of subarachnoid hemorrhage (SAH) are challenging to conduct for a number of reasons, such as relatively young age at diagnosis, low overall incidence of disease, and high death-rate from the first bleed. The latter implies that outside-hospital deaths from SAH are comprehensively diagnosed only in countries with a high coverage of forensic autopsies for sudden deaths. The number of large, long-term, and population-based prospective studies on risk factors for SAH is limited.[1–3] According to these studies, the most important risk factors for SAH are smoking, hypertension, female sex, and increasing age.[1–3]

The role of total cholesterol (TC) in risk for SAH is conflicting, since studies report both high[3–6] and low[7–12] TC to raise risk. Meta-analyses[13–15] suggest no association between TC and SAH, whereas they imply that high HDL is protective against SAH. The reviews do not include, however, a number of studies, such as a recent large study suggesting that high TC levels (>6.22 mmol/l) associate with future SAH in men.[3] As blood lipid levels and especially high levels of low-density lipoprotein (LDL) are important risk factors for cardiovascular diseases in general,[16] we reviewed current evidence on the role of TC and lipoproteins as risk factors for SAH. In contrast to the preceding reviews,[13–15] ours also focuses in depth on methodological quality of the studies reviewed. Readers should note that M.K. and J.K. have published one of the studies reviewed. [3]

## Methods

### Literature search

The protocol of this study is available in PROSPERO (International prospective register of systematic reviews, registration code: CRD42015016347) and follows the Preferred Reporting Items for Systematic reviews and Meta-analyses for Protocols Statement (PRISMA-P). The authors J.V.L. and M.K. did all steps of the protocol below; discussion with third-author J.K. resolved disagreements. We performed the literature search in two parts using Cochrane Library, Pubmed, and Scopus databases with no language limitations “S1 Methods”. As we included non-English-language publications, native speakers assisted when necessary. We included all studies reporting effect estimates with a minimum of two categories for TC, LDL, HDL, or apolipoprotein concentrations. Depending on the design, we divided all studies into either prospective or retrospective to facilitate risk-of-bias evaluations. After reviewing risk-of-bias guidelines from the Cochrane Collaboration Handbook,[17] Critical Appraisal Skills Program (CASP),[18] Newcastle-Ottawa scale,[19] and a measurement tool for assessment of multiple systematic reviews (AMSTAR) [20]we based our risk-of-bias estimations to Cochrane Collaboration Handbook [17] and CASP [18].

We used the PRISMA checklist[21] as a guide to achieve the accepted standards for reporting systematic reviews. Based on the Cochrane Collaborations guidelines,[17] we classified risk of bias into high, unclear, and low-grade categories. Our review focused especially on measurement bias, selection bias, reporting bias, confounder adjustment, reverse causality, and statistical power.

### Statistical analysis

Based on the Cox proportional hazards model, we estimated sufficient sample size for prospective follow-up studies. We used a standard significance value of p<0.05, standard power value of P = 0.8, and incidence value of 20/100 000. For optimistic power analysis, we selected the hazard ratio (HR) 2.0 (95% CI 1.0–4.0) as effect size with a wide confidence interval and low correlation factor value of 0.1 between covariates. We used a random effects model to calculate pooled estimates and assessed heterogeneity with I^2^-statistics. For two studies,[7,22] we inverted the reference category in order to present comparable results. Population attributable risk (PAR) was calculated with following formula: PAR = p_f_ (RR-1)/[p_f_ (RR-1)+1], where p_f_ is population fraction with hypercholesterolemia. Stata statistical software version 12.1 Stata Corp performed all the calculations.

## Results

### General

#### Search results

**Fig 1:**
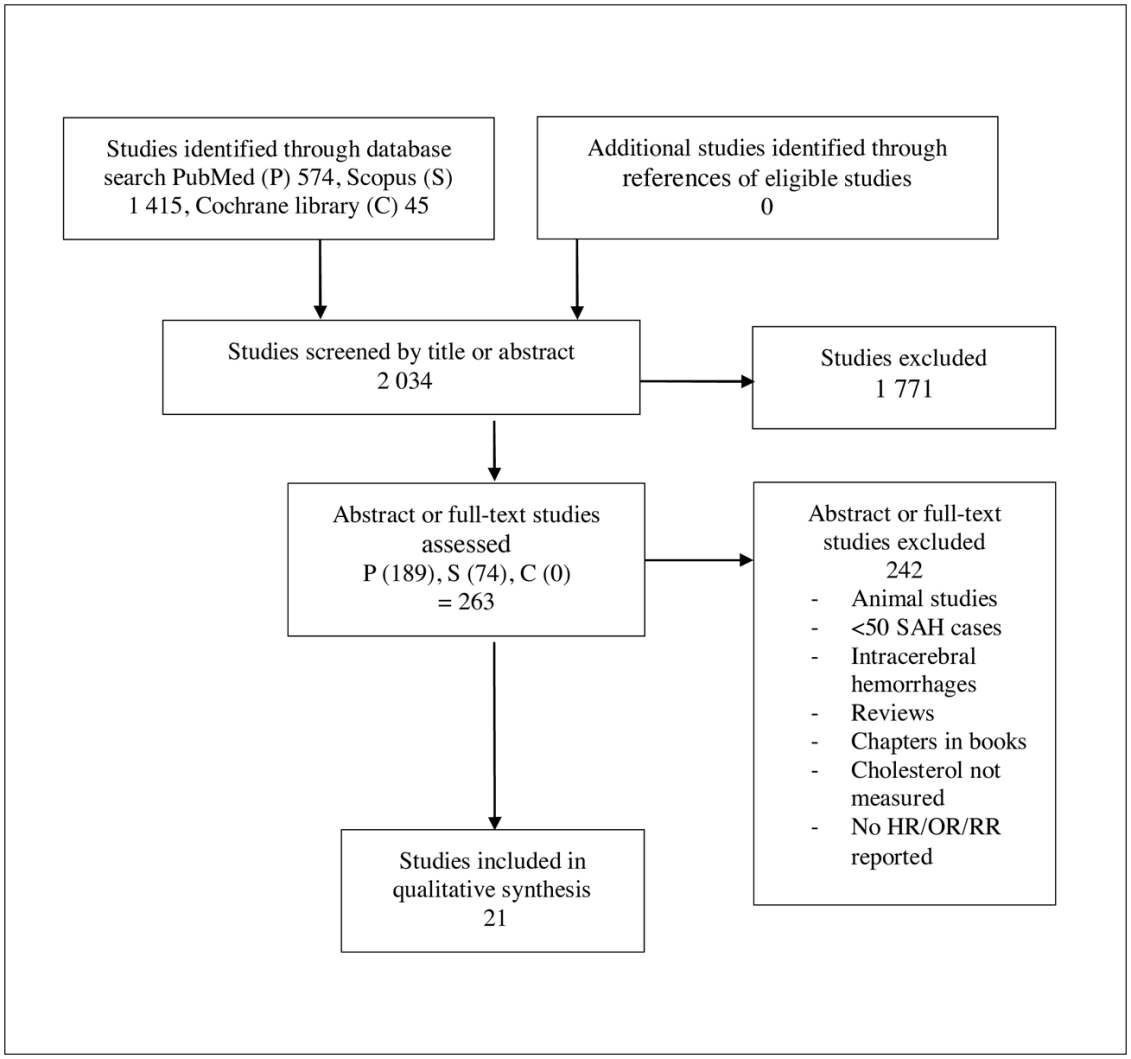
PRISMA Flow diagram. Study selection protocol.

In brief, 21 studies[1–12,22–30] including ≥50 SAH cases emerged that reported associations between risk for SAH and TC levels “Fig 1”.

#### Cohort characteristics

Cohort sizes and follow-up times in prospective studies differed considerably, and the number of SAH ranged from 55[28] to 437[3] (Table 1). In the retrospective studies, the number of SAHs ranged from 95[5] to 858.[9] The reported mean or median age at SAH in all studies ranged from 46.9[1] to 64[9] years (Table 1). Supplementary file “S1 Cohorts” describes the data collection in all 12 prospective studies in detail.

**Table 1:** Study characteristics. Summary of study characteristics and differences between prospective and retrospective studies.

Authors and Country	Year	Size	Age	% of men	Median/mean Follow-up	SAH cases	Fatal or non-fatal SAHs included	Outside-hospital deaths included	Median/mean age at SAH
**Prospective studies**									
**Finland**									
**Knekt** [1]	1991	42 862	20–69	54	12	187	Both	Yes	46.9
**Korja** [3]	2013	64 349	25–74	48.3	17.9	437	Both	Yes	59.3
**Leppälä** [29]	1999	28 519	50–69	100	6	85	Both	Yes	NR
**Zhang** [6]	2012	58 235	25–74	47.6	20.1	332	Both	Yes	NR
**Japan**									
**Cui** [27]	2007	38 158	40–79	38	10	66	Fatal	NR	61.9
**Suzuki** [8]	2011	156 892	30–89	48.7	3	223	Both	No	NR
**Norway**									
**Sandvei** [2]	2012	92 682	20–90+	33.6	10.9	122	Both	Yes	58.9
**South Korea**									
**Suh** [22]	2001	114 793	35–59	100	6	98	Both	No	NR
**Sweden**									
**Gatchev** [7]	1993	54 385	25–74	40.1	20.5	87	Fatal	NR	NR
**USA**									
**Iso** [28] *	1989	350 977	35–57	100	6	55	Fatal	No	47.4
**Neaton** [24] *,†	1993	353 340	35–57	100	25	259	Fatal	No	47.2
**Tirschwell** [30]	2004	8 010	30–79	44.2	11	96	Both	NR	N/A‡
**Retrospective**									
**Great Britain & Denmark**									
**Adamson** [25]	1994	189	25–70	44.8	N/A	96	NR	No	48
**Japan**									
**Inagawa** [4]	2005	494	25–96	43.7	N/A	247	Both	No	62.6
**Inagawa** [9]	2010	1 941	31–89	35	N/A	858	Both	NR	64
**Ohkuma** [10]	2003	780	25–85≤	31	N/A	390	NR	No	58
**Tokuda** [11]	2005	300	24–95	29	N/A	150	NR	No	60
**Holland**									
**Vlak** [12]	2013	824	54.8 (15.8)§	29	N/A	250	Non-fatal	No	54.7
**Portugal**									
**Canhao** [26]	1994	423	<40–65<	30.5	N/A	141	NR	NR	NR
**South Korea**									
**Park** [5]	1998	464 II	21–86	48.1	N/A	95	Both	NR	51.7
**USA**									
**Broderick** [23]	2003	930	19–49	39	N/A	312	Non-fatal	No	NR

*90.1% European. 6.4% African-American. 4.5% Hispanic or Oriental origin

^†^Includes all SAH deaths through 1999 as additional data

^‡^ Mean reported only for hemorrhagic stroke category

^§^ Standard deviation

II 102 intra cranial hemorrhages included in the number

NR = not reported. N/A = not applicable

#### Cases

Eight prospective[1–3,6,8,22,29,30] and three[4,5,9] retrospective studies comprised both fatal and none-fatal SAHs. Five[1–3,6,29] prospective studies (all Finnish or Norwegian) also reported inclusion of outside hospital deaths (Table 1). Diagnosis-verification protocol (computed tomography or lumbar puncture) was sufficient in all studies.

### Total cholesterol

#### Associations in prospective studies

Of the 12 studies,[1–3,6–8,22,24,27–30] two[7,8] suggested that low TC elevated risk for SAH (Table 2). The Swedish study observed, without having data on smoking, that low TC in men elevated SAH risk with a relative risk (RR) of 3.43 (Table 2).[7] The Japanese hospital-based cohort study reported that high TC lowered SAH risk with varying RRs 0.2–0.5 in low vs high analysis (no dose-response reported) including men and women with all their values above 4.14 mmol/l (Table 2).[8]

**Table 2:** Associations between TC and SAH. Associations between TC levels and SAH, number of SAHs in the subgroup analyses, and type of TC measurement with control group.

Authors and country	Lowest vs highest or vice versa (mmol/l)	HR/RR/OR and 95% CIs	No. of SAHs among men	No. of male cases in highest category	No. of SAHs among women	No. of female cases in highest category	Cases´ TC measurement and type of cohort or control group
**Prospective**							
**Finland**							
**Knekt** [1]	≤5.96 vs 6.99<	M 0.9 (0.6–1.5) W 1.0 (0.6–1.8)	102	39	85	39	sB, PB
**Korja** [3]	<4.92 vs 7.07<	M 2.18 (1.19–4.00) W 0.99 (0.62–1.59)	206	58	231	60	sB, PB
**Leppälä** [29]	<4.90 vs 7.00≤	0.78 (0.38–1.62)	85	19	-	-	B, MS
**Zhang** [6]	<5.00 vs 7.00<	M 1.79 (1.00–3.19) W 1.25 (0.71–2.20)	151	49	181	46	sB, PB
**Japan**							
**Cui** [27]	<4.14 vs 7.22≤	0.60 (0.08–4.73)	25	NR	41	NR	nB, PB
**Suzuki** [8]	<4.14 vs 7.24≤	0.2 (0.0–0.8)	89	NR	134	NR	B, H
**Norway**							
**Sandvei** [2]	5.9, per SD (1.3)	0.9 (0.7–1.1)	41	NR	81	NR	nB, PB
**South Korea**							
**Suh** [22] §	<4.31 vs 5.70<	0.69 (0.37–1.32)	98	18	-	-	fB, CP
**Sweden**							
**Gatchev** [7] §	M 5.3 vs 7.6 W 5.4 vs 7.9	M 0.29 (0.10–0.89) W 0.98 (0.42–2.08)	37	4	50	12	nB, PB
**USA**							
**Iso** [28]	<4.14 vs 7.24≤	0.16 *	55	1	-	-	nB, IG
**Neaton** [24]	<4.14 vs 7.24≤	1.70 (0.76–3.83)	259	17	-	-	nB, IG
**Tirschwell** [30]	4.45 vs 7.50 †	1.3 (0.7–2.4)	NR	NR	NR	NR	B, HM
**Retrospective**							
**Great Britain & Denmark**							
**Adamson** [25]	<5.40 vs 6.30<	15.7 (2.8–89)	43	NR	53	NR	aB, HE
**Japan**							
**Inagawa** [4]	<5.69 vs 5.69<	M 2.18 (0.67–7.08) W 4.08 (1.59–10.46)	108	18	139	48	aB, HE
**Inagawa** [9]	<5.69 vs 5.69<	M 0.89 (0.50–1.60) W 0.73 (0.55–0.98)	276	NR	522	NR	aB, HC
**Ohkuma** [10]	NR	0.41 (0.24–0.71)	119	NR	271	NR	I, HE
**Tokuda** [11]	<5.20 vs 5.20<	0.22 (0.12–0.40)	43	NR	107	NR	aB, H
**Holland**							
**Vlak** [12]	NR	0.2 (0.1–0.4)	62	NR	188	NR	I, HC
**Portugal**							
**Canhao** [26]	<6.21 vs 6.21<	0.9 (0.5–1.7)	95	27	46	67	I, HC
**Park** [5]	<3.88 vs 4.9<	1.02 (1.01–1.02)	24	NR	71	NR	aB, A
**USA**							
**Broderick** [23]	NR	0.53	NR	NR	NR	NR	I, HE

M = men, W = women, A = acute patients, B = blood sample with no fasting information, aB = blood sample on admission, fB = fasting blood sample, nB = non-fasting blood sample, sB = semi-fasting blood sample, CP = male civil service and private school workers, H = hospital based, HC = health check-up, HE = healthy, HM = hypertensive men and menopausal women, I = interview, IG = industry-company workers or government staff, MS = male smokers, PB = population based, NR = not reported

*Not significant and CIs not provided

^†^ Quintile means

^§^ Reference group inverted in studies of Gatchev and Suh

Two population-based studies,[3,6] including also outside hospital deaths, found that elevated TC levels (>5.59 mmol/l, >6.22 mmol/l, and ≥7.07 mmol/l vs ≤4.92 mmol/l[3] and ≥7.0 mmol/l vs <5.0 mmol/l[6]) increased risk for SAH in men with an HR of 1.97[3], 1.85[3], 2.18[3], and 1.79[6] (Table 2). The other study also reported dose response as a 1 mmol/l increase in TC raised risk for SAH in men with an HR of 1.16.[6] The remaining eight prospective studies[1,2,22,24,27–30] found no associations between TC and SAH (Table 2).

#### Associations in retrospective studies

Of the nine[4,5,9–12,23,25,26] studies, four[9–12] found that high TC measured by interview[10,12] or on admission (with hospital-based or health check-up control group),[9,11] decreased SAH risk with ORs of 0.2 [12], 0.22 [11], 0.41 [10] and (in women only) 0.73 (Table 2).[9]

Three[4,5,25] studies with on-admission measurements found that elevated TC (>4.9[5], >5.7[4], >6.3[24] mmol/l) elevated risk for SAH (ORs 1.02[5], 2.4[4], 15.7[24]) (Table 2). One of these studies also found an increased risk for SAH in women with high TC (>5.7 mmol/l) with an RR of 4.08 (Table 2).[4] Two[4,25] of these three[4,5,25] used mainly healthy controls, and one[5] used other acute emergency room patients and their on-admission TC values (Table 2). Two[23,26] studies found no associations between TC and SAH (Table 2).

#### TC range and power in prospective studies

Nine studies[2,3,6,8,24,27–30] included a wide range of TC values, but only three[1,3,6] reported a sufficient sample size (≥31) of male and female SAHs in the high-TC group to determine an HR under 2 (Tables 2 and 3). Table 2 and “S1 Table” present TC measurement protocols in detail.

**Table 3:** Sample sizes. Required number of person-years and SAHs for subgroup analysis for different HR values.

HR	SAH in subgroup studied	Person years needed
1.5	91	452 854
1.6	68	337 026
1.7	53	264 414
1.8	44	215 490
1.9	37	180 715
2.0	31	154 958
2.1	28	135 248
2.2	24	119 760
2.3	22	107 318
2.4	20	97 137
2.5	18	88 675
2.6	17	81 545
2.7	16	75 466
2.8	15	70 229

p<0.05, P = 0.8, covariate correlation factor 0.1, and incidence 20/100 000

#### TC measurement and range in retrospective studies

Five[4,5,9,11,25] studies measured TC on admission after SAH and four[10,12,23,26] by interview. None of the nine[4,5,9–12,23,25,26] studies included a wide range of TC values in their analysis (Table 2).

#### Risk-factor adjustments in prospective studies

All[1–3,6,8,22,24,27–30] except one study[7] recorded known SAH risk factors: age, sex, hypertension, and smoking (or comprised only smokers or one sex), and all but two[2,7] controlled for them in a multivariable model. One study reporting high[6] and one low[8] TC as elevating risk for SAH also controlled for BMI values for confounding in analyses. The two studies reporting low TC as elevating risk lacked data on smoking[7] or analysis by sex.[8]

#### Risk-factor adjustments in retrospective studies

All nine[4,5,9–12,23,25,26] studies recorded known risk factors for SAH, and the four[9–12] reporting low and the three[4,5,25] high TC as a risk factor for SAH controlled for them all.

#### Low-TC studies

Five retrospective[4,5,9–11] and three[8,22,27] prospective studies came from Japan and South Korea, where TC levels are generally lower than in Western countries. Separate analysis of these studies was still conflicting, since two[4,5] reported high and four[8–11] low TC as elevating risk, and two[22,27] remained inconclusive (Table 2).

#### Brief summary of TC findings

Of the 12[1–3,6–8,22,24,27–30] prospective studies, 2[3,6] found high TC raising risk for SAH in men. Conversely, two prospective studies found low TC as raising risk for SAH in men[7] and in both men and women[8] while eight[1,2,22,24,27–30] were indecisive (Table 2). Of the nine[4,5,9–12,23,25,26]retrospective studies, four[9–12] found low and three[4,5,25] high TC as raising risk for SAH whereas the others[23,26] remained inconsistent.

### Subfractions of cholesterol

#### HDL and other lipoproteins

Three[2,6,29] prospective and one[25] retrospective study analyzed associations between HDL and risk for SAH (Table 4). The one[25] retrospective also analyzed association of apolipoprotein B (ApoB) with SAH. None of the studies focused on any other lipoprotein subtypes, such as LDL, or the lipid subfractions nowadays available with lipidomic analyses.[31] S1 Table presents a detailed summary of different laboratory methods that studies used to analyze HDL.

**Table 4:** Associations between HDL and SAH. Associations between HDL levels and SAHs, number of SAHs in subgroup analyses and type of HDL measurement with control group.

Authors and Country	Protective factor	Highest vs lowest (mmol/l)	HR/RR/OR and 95% CIs	No. of SAHs among men	No. of male cases in lowest category	No. of SAHs among women	No. of female cases in lowest category	Cases´ TC measurement and type of controls or cohort
**Prospective**								
**Finland**								
**Leppälä** [29]	High HDL	1.45< vs <0.84	0.26 (0.11–0.62)	85	14	0	0	B, SM
**Zhang** [6]	None	<1.0 vs 1.4<	M 0.56 (0.25–1.25) W 1.27 (0.50–3.28)	151	17	181	15	sB, PB
**Norway**								
**Sandvei** [2]	None	1.4 (SD 0.4)	1.0 (0.9–1.3)*	41	NR	81	NR	nB, PB
**<50 years**	High HDL	1.4 (SD 0.4)	0.6 (0.4–0.9)*	NR	NR	NR	NR	
**>50 years**	Low HDL	1.4 (SD 0.4)	1.2 (1.0–1.5)*	NR	NR	NR	NR	
**Retrospective**								
**Great Britain & Denmark**								
**Adamson** [25]	None	1.4< vs <1.1	NR	43	NR	53	NR	aB, HE
**ApoB (g/l)**	None	0.83< vs <0.65	1.0 CIs NR	43	NR	53	NR	

M = men, W = women, B = unknown blood sample, aB = on admission blood sample, nB = non-fasting blood sample, sB = semi-fasting blood sample, HE = healthy controls, PB = population based cohort, SM = cohort of male smokers, NR = not reported

* per SD increase

#### Associations

One prospective study reported that risk for SAH decreased with an HR of 0.6 per SD unit increase in HDL in the subgroup under age 50 when they adjusted for age, sex, smoking, and alcohol consumption, but not for hypertension (Table 4).[2] Another prospective study controlled for known risk factors and also excluded participants who were using cholesterol-lowering medication.[6] After exclusion, however, the association between HDL and SAH remained unchanged. One study, comprising only male smokers, reported a decreasing risk for SAH with increasing HDL values (≥0.84 mmol/l) in their prospective setup (Table 4).[29] The only retrospective study focusing on lipoprotein subtypes used on-admission measurements, controlled for known risk factors for SAH, and found no significant associations between HDL, ApoB, and SAH [25] (Table 4).

### Risk of bias and power analyses

#### Prospective studies

**Fig 2:**
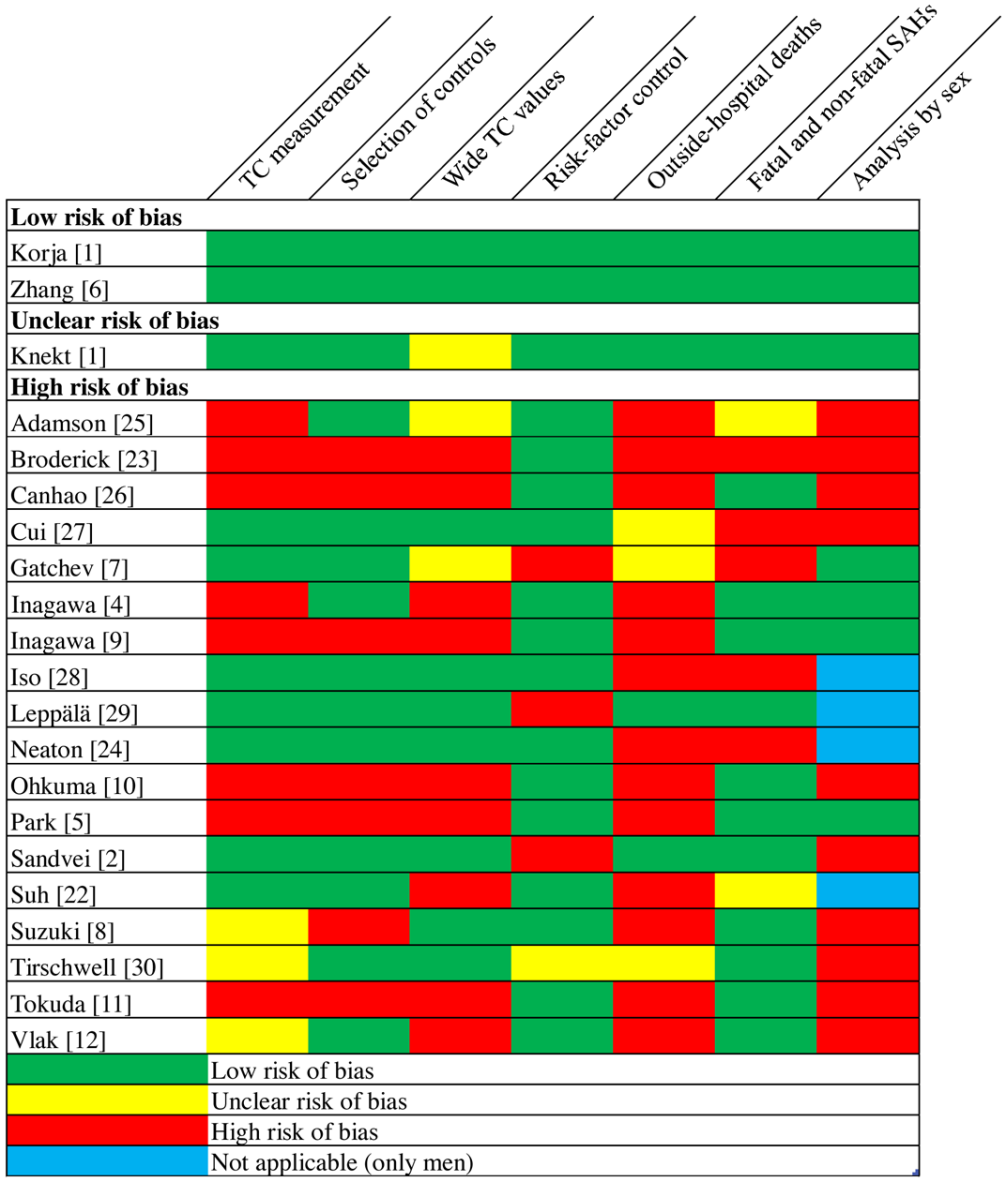
Risk of bias. Classification of risk of bias and sources of bias in all studies.

Of the 12[1–3,6–8,22,24,27–30] studies, only 2[3,6] had a low risk of bias; those 2 had a sufficient number of SAHs, included outside hospital sudden deaths, comprehensive risk factor control, analysis by sex, wide TC range, unlikely selection bias, sufficient accuracy (validation) of diagnosis, unlikely measurement bias, and low risk for reverse causality “Fig 2”. In addition, although one[1] study met these criteria, all participants had very high TC levels; we thus determined that it had an unclear risk of bias. Nine studies[2,7,8,22,24,27–30] had a high risk of bias because of limitations in the aforementioned criteria “Fig 2”. Moreover, power calculations suggested that only three[1,3,6] studies were able to detect significant associations between high TC and SAH (Table 3).

#### Retrospective and low-TC studies

All nine[4,5,9–12,23,25,26] retrospective and eight[4,5,8–11,22,27] low-TC studies had a high risk of bias because of limitations in the aforementioned criteria “Fig 2”.

#### Small studies

Since small studies tend to combine SAH and intracranial hemorrhage in a group of hemorrhagic stroke, we excluded them from the analysis.

#### Studies on HDL

Of the four[2,6,25,29] studies, only one[6] had a low risk of bias, because it met the aforementioned criteria.

#### Meta-analysis

In addition to evident methodological differences among studies “Fig 2”, our meta-analysis showed also considerable heterogeneity. Heterogeneity among retrospective and Asian studies was high (I^2^ = 79.5%-89.0%), and considerable in prospective studies (I^2^ = 46.0%) “Figures A-D in S1 Figures”. Indisputably, meta-analysis on reviewed studies lacks justification. However, we performed a series of sensitivity analyses and selected the most similar studies for meta-analysis.

A meta-analysis limited to prospective studies with comparable wide TC groups, major limitations only in an analysis by sex and exclusion of certain SAH types,[3,24,27,30] showed moderate evidence that high TC elevates SAH risk with an RR of 1.33 “Figure E in S1 Figures”.

The meta-analysis including only retrospective studies with TC values measured in an acute phase[9,11,25] showed no association between TC and SAH “Figure F in S1 Figures”. Meta-analysis including only studies which obtained TC values using interviews[10,12,26] showed low TC as risk factor (RR 0.42) “Figure G in S1 Figures”. The sensitivity analysis included a small number of studies, and has unreliable heterogeneity estimates.

## Discussion

Some relatively high-quality evidence[3,6] suggests that high TC is a risk factor for SAH in men questioning the current understanding of SAH epidemiology. The role of HDL remained unresolved. [6] Only one[25] retrospective study focused on associations between ApoB and SAH, but found none, whereas none studied LDL. Meta-analyses[13–15] did not find strong associations between SAH and TC. However, they did not include extensive risk-of-bias analyses and, therefore may have failed to uncover sources of heterogeneity in the studies included.

Internal quality of SAH risk factor studies varies considerably, as most have significant methodological shortages and gaps in reporting. Five studies[3–6,25] reported high TC, six[7–12] low TC, and ten others[1,2,22–24,26–30] neither one as increasing risk for SAH. Of all 12[1–3,6–8,22,24,27–30] prospective studies, 3[1,3,6] had sufficient statistical power to detect associations between high TC and SAH. Of all 21 studies,[1–12,22–30] only 2[3,6] had a low risk of bias. According to the classical Hills criteria of causality,[32] the two studies also had convincing temporality, biological gradient, plausibility, coherence, and analogy. These studies also included outside hospital SAH deaths. Both originated from the FINNRISK cohort but from different study groups. The most recent[3] included more SAH cases and found stronger associations between elevated TC and SAH. Also sensitivity analysis including prospective studies (with comparable TC levels and only few limitations) supported the view that elevated TC associates with SAH risk. As TC is a well-known risk factor for cardiovascular diseases in general,[16] and atherosclerotic changes are commonly found in aneurysm walls,[33] the results for TC seem reasonable. Analysis of low-TC studies separately provided no support for the hypothesis of a U-shaped risk curve.

The recent low-risk-of-bias study suggested[3] that TC levels over 5.6mmol/l elevate risk of SAH with HR of approximately 2 in men. Based on the varying prevalence (between 17% and 55%) of hypercholesterolemia worldwide,[34] the HR of 2 in men translates into PAR estimates ranging from 17% to 35%. The PAR of hypercholesterolemia in men can reach up to 35% in Europe, and up to 32% in the USA. Because TC levels are increasing in a number of countries,[35] PAR of hypercholesterolemia is likely to increase especially in developing countries. The recent low-risk-of-bias study reported HRs of 2.53 and 1.23 for smoking and hypertension in men. [3] With a prevalence estimate of 25% for smoking and 41% for hypertension, the HRs translate into PARs of 28% and 9% in men. Thus, TC may significantly contribute to the SAH incidence among men, and to the difference in risk-factor profiles between sexes.

TC and LDL levels are well-documented risk factors for other cardiovascular diseases (CVDs), whereas HDL is generally considered a protective risk indicator.[16] The role of a high LDL is considered to be causal, whereas the causal nature of the HDL association has been questioned.[36–38] Furthermore, the influence of these factors on CVDs may differ by sex[39,40] because premenopausal women are also at lower risk for CVDs than are age-matched men.[16] Physiological changes during menopause elevate risk for CVDs,[41] when TC and LDL levels start to rise at its beginning and reach their peak at approximately age 60.[39] At the same time, HDL decreases and triglycerides increase, making the lipid profile more unfavorable, thus elevating risk for CVDs, especially in women.[39] Of those included, two[3,8] prospective studies reporting age-group SAH incidences, further stratified by sex, showed SAH incidence in women to surpass male incidence at age 65 and above. TC levels often being low in premenopausal women may in part explain why associations between TC and SAH are difficult to discover. This emphasizes the importance of analyzing and representing TC associations by sex and age and indicates that future SAH studies should focus on the role of TC in both pre- and postmenopausal women separately.

Prospective follow-up studies require a significant number (approx. 270 000) of person-years and optimally over 35 SAHs for each sex per TC subgroup to reliably study associations between TC levels and risk for SAH. In addition, because roughly 20% of SAH patients die away from hospitals or in emergency rooms,[42] and are thus excluded from most studies, even more person-years may be necessary. Only four[1,3,6,29] Finnish and one Norwegian[2] population-based studies reported in their analyses inclusion of sudden deaths. Furthermore, TC values should be analyzed as a continuous variable and should include adequate numbers of participants with TC below 5.0 mmol/l and above 6.5 mmol/l. As the median age at SAH is nowadays 60 years,[2,3] the study cohort should be of a rather high median age with a very long follow-up. Even then, reliable studies on TC are nowadays very challenging to conduct, as a high proportion of those with hypercholesterolemia use statins. A recent retrospective study [43] relying on administrative data suggested that statin use in elderly patients with unruptured intracranial aneurysms does not affect the incidence rate of SAH. However, the study did not report TC levels at baseline or after statin treatment, and did not stratify the results by sex. All these factors considered, it is clear that discovering the epidemiological role of TC in SAH is challenging; relatively small and short-term studies need cautious interpretation.

One of the most important shortcomings in the prospective cohort studies reviewed is their lack of longitudinal follow-up data; changes in risk-factor profile during the study period may therefore distort and confound association analyses. For example, in prospective studies from Norway[2] and Finland,[3,6] TC levels were measured 6 (mean) and 12 (median) years before SAH. As TC values have been a target of intensive drug and diet therapies during recent decades,[16] it is likely that baseline values had changed during follow-ups. Indeed, since the 1970s, when the study surveys began, the decline in TC of the Finnish population has been rapid, [44] and thus the risk estimates for TC levels may be unreliable. However, these modifications can only underestimate the role of high TC levels as a risk factor for SAH, making any associations between TC and SAH only decrease or disappear.

The reliability of retrospective studies in finding new risk factors for SAH is generally limited, not only because all outside hospital deaths are excluded from analyses, but also because of the evident risk of measurement bias when risk factors are measured by interview or after SAH. This was also evident in our sensitivity analyses, which included only on admission TC measurements or measurements by interview. Studies which measured TC by interview suggested that low TC elevates SAH risk. As TC levels may decrease during and after SAH,[45,46] all studies which measure TC on admission or within days after SAH are prone to reverse causality. This view is consistent with the results of the retrospective study[9] reporting TC as a risk factor for aneurysm formation but not for SAH. In addition, selection bias may weaken the generalizability of hospital-based studies by limiting the inclusion of patients with low TC in their control groups. Indeed, those retrospective studies[4,5,25] that reported high TC as leading to increased risk for SAH, used other emergency room patients or healthy people as control groups.

Our review has a few shortcomings. First, we may have missed some relevant publications due to indexing errors and limitations in the search strategy. Our search strategy, however, yielded 21 reviewed studies whereas the three earlier systematic reviews on this topic comprised only 7,[15] 6,[13] and 3[14] studies with over 50 SAH cases. Second, the review includes study by two authors (M.K. and J.K.), making it possible that unintentional conflict of interest has biased risk-of-bias estimations. We tried to avoid this with an established and public study protocol registered in the PROSPERO. Despite this, readers need to take this into consideration when evaluating the presented evidence. Third, a reliable meta-analysis, one which could have elucidated the role of TC in SAH, was impossible due to methodological differences and lack of the necessary additional data.

## Conclusion

Elevated TC levels may elevate risk for SAH among men, and therefore lipid profile should perhaps be taken into account when assessing risk for SAH in men. No convincing evidence exist that low TC elevates risk for SAH. Apart from diabetes and obesity, the risk-factor profile of SAH resembles that of other CVDs, at least in men.

## Supporting Information

S1 Cohorts Description of cohorts used in prospective studies. (DOCX)

S1 Figures Meta-analysis and sensitivity analyses. (DOCX)

S1 Methods Search strategy and protocol. (DOCX)

S1 PRISMA Checklist (DOC)

S1 Table Cholesterol measurement protocols used in studies reviewed. (DOCX)
