# Supplementary material for: Cholesterol as a Risk Factor for Subarachnoid Hemorrhage: A Systematic Review
Source: PLoS One. 2016 Apr 14;11(4):e0152568. doi: 10.1371/journal.pone.0152568 (PMC4831795; doi:10.1371/journal.pone.0152568)
Supplement: S1 Cohorts — (DOCX) [file pone.0152568.s001.docx]

Supplementary File Cohort

Cohort characteristics

*Data collection in 12 prospective cohorts*. The Swedish study collected baseline data between 1964 and 1965 that were based on a general health survey.[1] Of the four Finnish[2-5] studies, one[2] arose from a population-based multiphasic screening examination[6] done between 1966 and 1972, whereas two[3,4] were based on the same ongoing population-based FINNRISK study which started in 1972 with surveys every five years.[7] One Finnish study[5] came out of the Alpha-Tocopherol, Beta-Carotene Cancer Prevention (ATBC) study done between 1985 and 1988.[8] Of the three[9-11] studies from the USA, two[9,10] originated from the Multiple Risk Factor Intervention Trial (MRFIT) study, done between 1973 and 1975[12] and one[11] from the Group Health Cooperative (GCH) study, done between 1989 and 2000. Two studies came from Japan: one[13] was a nested case-control study stemming from a population-based Japan collaborative cohort study (JACC-study) done between 1988 and 1990,[14] whereas the other was a hospital-based mass screening study in Akita prefecture between 1991 and 1998.[15] The South Korean study,[16] based on the Korean Medical Insurance Corporation Study, was done between 1990 and 1992.[17] The Norwegian study collected data between 1994 and 1997,[18] and was based on two different population-based cohorts, namely the HUNT 2[19] and Tromsø 4.[20] In all studies, personnel advised participants with very high TC to contact their general practitioners.

Supplementary references

(1) Gatchev O, Rastam L, Lindberg G, Gullberg B, Eklund GA, Isacsson SO. Subarachnoid hemorrhage, cerebral hemorrhage, and serum cholesterol concentration in men and women. Ann Epidemiol 1993 Jul;3(4):403-409.

(2) Knekt P, Reunanen A, Aho K, Heliovaara M, Rissanen A, Aromaa A, et al. Risk factors for subarachnoid hemorrhage in a longitudinal population study. J Clin Epidemiol 1991;44(9):933-939.

(3) Korja M, Silventoinen K, Laatikainen T, Jousilahti P, Salomaa V, Hernesniemi J, et al. Risk factors and their combined effects on the incidence rate of subarachnoid hemorrhage - a population-based cohort study. PLoS One 2013 Sep 9;8(9):e73760.

(4) Zhang Y, Tuomilehto J, Jousilahti P, Wang Y, Antikainen R, Hu G. Total and high-density lipoprotein cholesterol and stroke risk. Stroke 2012 Jul;43(7):1768-1774.

(5) Leppala JM, Virtamo J, Fogelholm R, Albanes D, Heinonen OP. Different risk factors for different stroke subtypes: association of blood pressure, cholesterol, and antioxidants. Stroke 1999 Dec;30(12):2535-2540.

(6) Aromaa A. Aromaa A. Epidemiology and Public Health Impact of
High Blood Pressure in Finland. (In Finnish with
English summary) Helsinki: Publications of the Social
Insurance Institution, Finland, AL: 17, 1981. 1981.

(7) Borodulin K, Vartiainen E, Peltonen M, Jousilahti P, Juolevi A, Laatikainen T, et al. Forty-year trends in cardiovascular risk factors in Finland. Eur J Public Health 2015 Jun;25(3):539-546.

(8) The effect of vitamin E and beta carotene on the incidence of lung cancer and other cancers in male smokers. The Alpha-Tocopherol, Beta Carotene Cancer Prevention Study Group. N Engl J Med 1994 Apr 14;330(15):1029-1035.

(9) Iso H, Jacobs DR,Jr, Wentworth D, Neaton JD, Cohen JD. Serum cholesterol levels and six-year mortality from stroke in 350,977 men screened for the multiple risk factor intervention trial. N Engl J Med 1989 Apr 6;320(14):904-910.

(10) Neaton JD, Wentworth DN, Cutler J, Stamler J, Kuller L. Risk factors for death from different types of stroke. Multiple Risk Factor Intervention Trial Research Group. Ann Epidemiol 1993 Sep;3(5):493-499.

(11) Tirschwell DL, Smith NL, Heckbert SR, Lemaitre RN, Longstreth WT,Jr, Psaty BM. Association of cholesterol with stroke risk varies in stroke subtypes and patient subgroups. Neurology 2004 Nov 23;63(10):1868-1875.

(12) Multiple risk factor intervention trial. Risk factor changes and mortality results. Multiple Risk Factor Intervention Trial Research Group. JAMA 1982 Sep 24;248(12):1465-1477.

(13) Cui R, Iso H, Toyoshima H, Date C, Yamamoto A, Kikuchi S, et al. Serum total cholesterol levels and risk of mortality from stroke and coronary heart disease in Japanese: the JACC study. Atherosclerosis 2007 Oct;194(2):415-420.

(14) Ohno Y, Tamakoshi A, JACC Study Group. Japan collaborative cohort study for evaluation of cancer risk sponsored by monbusho (JACC study). J Epidemiol 2001 Jul;11(4):144-150.

(15) Suzuki K, Izumi M, Sakamoto T, Hayashi M. Blood pressure and total cholesterol level are critical risks especially for hemorrhagic stroke in Akita, Japan. Cerebrovasc Dis 2011;31(1):100-106.

(16) Suh I, Jee SH, Kim HC, Nam CM, Kim IS, Appel LJ. Low serum cholesterol and haemorrhagic stroke in men: Korea Medical Insurance Corporation Study. Lancet 2001 Mar 24;357(9260):922-925.

(17) Jee SH, Appel LJ, Suh I, Whelton PK, Kim IS. Prevalence of cardiovascular risk factors in South Korean adults: results from the Korea Medical Insurance Corporation (KMIC) Study. Ann Epidemiol 1998 Jan;8(1):14-21.

(18) Sandvei MS, Lindekleiv H, Romundstad PR, Muller TB, Vatten LJ, Ingebrigtsen T, et al. Risk factors for aneurysmal subarachnoid hemorrhage - BMI and serum lipids: 11-year follow-up of the HUNT and the Tromso Study in Norway. Acta Neurol Scand 2012 Jun;125(6):382-388.

(19) Krokstad S, Langhammer A, Hveem K, Holmen TL, Midthjell K, Stene TR, et al. Cohort Profile: the HUNT Study, Norway. Int J Epidemiol 2013 Aug;42(4):968-977.

(20) Jacobsen BK, Eggen AE, Mathiesen EB, Wilsgaard T, Njolstad I. Cohort profile: the Tromso Study. Int J Epidemiol 2012 Aug;41(4):961-967.
