# Supplementary material for: Cholesterol as a Risk Factor for Subarachnoid Hemorrhage: A Systematic Review
Source: PLoS One. 2016 Apr 14;11(4):e0152568. doi: 10.1371/journal.pone.0152568 (PMC4831795; doi:10.1371/journal.pone.0152568)
Supplement: S1 Figures — (DOCX) [file pone.0152568.s002.docx]

**Supplemental Figures**

Overall (I^2^=79.5%, p<0.001)

Gatchev M [7] ‡

Cui [27] †

Inagawa W [9]

Sandvei [2] §

Gatchev W [7] ‡

Korja M [3]

Park [5]

Suzuki [8]

Study*

Ohkuma [10]

Adamson[25]

Knekt M [1]

Canhao[26]

Knekt W [1]

Inagawa M [4]

Inagawa W [4]

Suh M [22] ‡

Inagawa M [9]

Korja W [3]

Tirschwell [30]

Neaton M [24]

Leppälä M [29]

Vlak [12]

Tokuda [11]

0.85 (0.69, 1.04)

0.29 (0.10, 0.89)

0.75 (0.24, 2.33)

0.73 (0.55, 0.98)

0.81 (0.66, 0.99)

0.98 (0.42, 2.08)

2.18 (1.19, 4.00)

1.02 (1.01, 1.02)

0.20 (0.04, 0.80)

0.41 (0.24, 0.71)

15.70 (2.80, 89.00)

0.90 (0.60, 1.50)

0.90 (0.50, 1.70)

1.00 (0.60, 1.80)

2.18 (0.67, 7.08)

4.08 (1.59, 10.46)

0.69 (0.37, 1.32)

0.89 (0.50, 1.60)

0.99 (0.62, 1.59)

1.30 (0.70, 2.40)

1.70 (0.76, 3.83)

0.78 (0.38, 1.62)

0.20 (0.10, 0.40)

0.22 (0.12, 0.40)

100.00

2.46

2.33

6.87

7.38

3.62

Weight

4.71

7.94

1.53

5.13

1.21

5.71

4.67

5.08

2.21

2.99

4.53

4.87

5.62

4.65

3.58

4.01

4.19

4.74

RR (95% CI)

.125

.2

.5

1

2

5

8

**Supplemental Figure A RRs in all studies**

Low TC is a risk factor

High TC is a risk factor

The horizontal lines show 95% CIs and the arrow indicates extension of the interval. Boxes show estimated RRs, and box size indicates the inverse variance weight of respective studies from the random effects analysis. The diamond shows a pooled RR and 95% CIs.

M=men, W=women.

Studies by Zhang and Iso are excluded since studies by Korja and Neaton are updated studies from the same cohorts.

^*^Under the assumption that Cox model coefficients, log relative risk estimates and log odds ratios are comparable.

†Converted to RR due to a small number of participants.

‡Reference group inverted.

§ per 2 SD

Overall (I^2^=46.0%, p=0.035)

Gatchev W [7] ‡

Korja M [3]

Gatchev M [7] ‡

Study*

Leppälä M [29]

Neaton M [24]

Sandvei [2] §

Korja W [3]

Suzuki [8]

Tirschwell [30]

Knekt M [1]

Cui [27] †

Knekt W [1]

Suh [22] ‡

0.93 (0.74, 1.18)

0.98 (0.42, 2.08)

2.18 (1.19, 4.00)

0.29 (0.10, 0.89)

RR (95% CI)

0.78 (0.38, 1.62)

1.70 (0.76, 3.83)

0.81 (0.66, 0.99)

0.99 (0.62, 1.59)

0.20 (0.04, 0.80)

1.30 (0.70, 2.40)

0.90 (0.60, 1.50)

0.75 (0.24, 2.33)

1.00 (0.60, 1.80)

0.69 (0.37, 1.32)

100.00

5.81

8.32

3.60

Weight %

6.65

5.72

17.22

10.81

2.09

8.16

11.08

3.38

9.29

7.86

High TC is a risk factor

Low TC is a risk factor

1

.125

.2

.5

2

5

8

**Supplemental Figure B RRs in prospective studies**

The horizontal lines show 95% CIs and the arrow indicates extension of the interval. Boxes show estimated RRs, and box size indicates the inverse variance weight of respective studies from the random effects analysis. The diamond shows a pooled RR and 95% CIs.

M=men, W=women.

Studies by Zhang and Iso are excluded since studies by Korja and Neaton are updated studies from the same cohorts.

^*^Under the assumption that Cox model coefficients, log relative risk estimates and log odds ratios are comparable.

†Converted to RR due to a small number of participants.

‡Reference group inverted.

§ per 2 SD

Overall (I^2^= 89.0%, p<0.001)

Inagawa M [4]

Ohkuma [10]

Tokuda [11]

Inagawa W [4]

Inagawa W [9]

Inagawa M [9]

Vlak [12]

Adamson [25]

Park [5]

Study

Canhao [26]

0.81 (0.53, 1.24)

2.18 (0.67, 7.08)

0.41 (0.24, 0.71)

0.22 (0.12, 0.40)

4.08 (1.59, 10.46)

0.73 (0.55, 0.98)

0.89 (0.50, 1.60)

0.20 (0.10, 0.40)

15.70 (2.80, 89.00)

1.02 (1.01, 1.02)

OR (95% CI)

0.90 (0.50, 1.70)

100.00

6.70

11.30

10.83

8.24

13.01

10.99

10.12

4.20

13.85

Weight %

10.76

Low TC is a risk factor

High TC is a risk factor

1

.125

.2

.5

2

5

8

**Supplemental Figure C ORs in retrospective studies**

The horizontal lines show 95% CIs and the arrow indicates extension of the interval. Boxes show estimated RRs, and box size indicates the inverse variance weight of respective studies from the random effects analysis. The diamond shows a pooled RR and 95% CIs.

Overall (I^2^= 84.3%, p<0.001)

Cui 2007 [27]

Inagawa 2010 W [9]

Study

Inagawa 2005 M [4]

Suzuki 2011 [8]

Suh 2001 [22]

Tokuda 2005 [11]

Inagawa 2010 M [9]

Park 1998 [5]

Ohkuma 2003 [10]

Inagawa 2005 W [4]

0.74 (0.50, 1.08)

0.60 (0.08, 4.73)

0.73 (0.55, 0.98)

2.18 (0.67, 7.08)

0.20 (0.04, 0.80)

0.69 (0.37, 1.32)

0.22 (0.12, 0.40)

0.89 (0.50, 1.60)

1.02 (1.01, 1.02)

0.41 (0.24, 0.71)

4.08 (1.59, 10.46)

100.00

2.93

14.78

Weight %

6.44

4.70

11.21

11.58

11.80

16.11

12.23

8.23

1

.125

.2

.5

2

5

8

**Supplemental Figure D ORs in Asian studies**

Low TC is a risk factor

High TC is a risk factor

OR (95% CI)

The horizontal lines show 95% CIs and the arrow indicates extensions of the interval. Boxes show estimated RRs, and box size indicates the inverse variance weight of respective studies from the random effects analysis. The diamond shows a pooled RR and 95% CIs.

Overall (I-squared = 25.8%, p = 0.249)

Korja W [3]

Study *

Tirschwell [30]

Korja M [3]

Low TC is a risk factor

Neaton M [24] *

Cui [27] †

1.33 (0.94, 1.88)

0.99 (0.62, 1.59)

1.30 (0.70, 2.40)

2.18 (1.19, 4.00)

RR (95% CI)

1.70 (0.76, 3.83)

0.75 (0.24, 2.33)

100.00

31.76

22.36

22.88

14.76

8.25

1.33 (0.94, 1.88)

0.99 (0.62, 1.59)

1.30 (0.70, 2.40)

2.18 (1.19, 4.00)

1.70 (0.76, 3.83)

0.75 (0.24, 2.33)

100.00

31.76

22.36

22.88

Weight %

14.76

8.25

1

.2

.5

1

2

5

**Supplemental Figure E RRs in similar prospective studies**

High TC is a risk factor

The horizontal lines show 95% CIs. Boxes show estimated RRs, and box size indicates the inverse variance weight of respective studies from the random effects analysis. The diamond shows a pooled RR and 95% CIs.

Studies by Zhang and Iso are excluded since studies by Korja and Neaton are updated studies from the same cohorts.

M=men, W=women.

*Under the assumption that Cox model coefficients and log relative risk estimates are comparable

†Converted to RR due to a small number of participants.

Overall (I-squared = 89.0%, p = 0.000)

Study

Inagawa M [9]

Tokuda [11]

Inagawa W [9]

Adamson [25]

0.87 (0.35, 2.15)

0.89 (0.50, 1.60)

0.22 (0.12, 0.40)

0.73 (0.55, 0.98)

15.70 (2.80, 89.00)

100.00

27.67

30.26

14.63

Weight %

0.87 (0.35, 2.15)

0.89 (0.50, 1.60)

0.22 (0.12, 0.40)

0.73 (0.55, 0.98)

RR (95% CI)

27.44

1

.2

.5

1

2

5

**Supplemental Figure F RRs in studies which measured TC from acute SAH patients**

Low TC is a risk factor

High TC is a risk factor

The horizontal lines show 95% CIs and the arrow indicates extension of the interval. Boxes show estimated RRs, and box size indicates the inverse variance weight of respective studies from the random effects analysis. The diamond shows a pooled RR and 95% CIs.

M=men, W=women.

Overall (I-squared = 80.6%, p = 0.006)

Study

Vlak [12]

Canhao [26]

Ohkuma [10]

0.42 (0.19, 0.95)

OR (95% CI)

0.20 (0.10, 0.40)

0.90 (0.50, 1.70)

0.41 (0.24, 0.71)

100.00

Weight %

31.69

33.43

34.88

100.00

31.69

33.43

34.88

1

.2

.5

1

2

5

**Supplemental Figure G ORs in studies which measured TC by interview**

Low TC is a risk factor

High TC is a risk factor

The horizontal lines show 95% CIs and the arrow indicates extension of the interval. Boxes show estimated RRs, and box size indicates the inverse variance weight of respective studies from the random effects analysis. The diamond shows a pooled RR and 95% CIs.
