# Supplementary material for: Cholesterol as a Risk Factor for Subarachnoid Hemorrhage: A Systematic Review
Source: PLoS One. 2016 Apr 14;11(4):e0152568. doi: 10.1371/journal.pone.0152568 (PMC4831795; doi:10.1371/journal.pone.0152568)
Supplement: S1 Methods — (DOCX) [file pone.0152568.s003.docx]

**Supplemental Methods 1**

**Search protocol**

**Pubmed 15.12.2015**

((((((("aneurysmal subarachnoid haemorrhage"[Title/Abstract]) AND risk[Title]))) OR (("aneurysmal subarachnoid hemorrhage"[Title/Abstract]) AND risk[Title]))) OR (((((intracranial aneurysm) OR cerebral hemorrhage) OR subarachnoid hemorrhage)) AND ((((hyperlipidemia) OR cholesterol)) AND risk factors))) OR (subarachnoid hemorrhage AND cholesterol)

Result 574

**Scopus 15.12.2015**

(((subara* AND (hemor* OR bleed* OR blo* OR vuo* OR blö*) OR *aneurysm*) AND (*cholest* OR *koles* OR *lipoprote* OR trigly* OR *HDL* OR *LDL*)) AND NOT DBCOLL(medl)) AND ( LIMIT-TO(SUBJAREA,"MEDI" ) OR LIMIT-TO(SUBJAREA,"NEUR" ) )

Result 1 415

**Cochrane library 15.12.2015**

(((((((aneurysmal subarachnoid haemorrhage) AND risk))) OR ((aneurysmal subarachnoid hemorrhage) AND risk))) OR (((((intracranial aneurysm) OR cerebral hemorrhage) OR subarachnoid hemorrhage)) AND ((((hyperlipidemia) OR cholesterol)) AND risk factors))) OR (Subarachniod hemorrhage AND cholesterol)

Results 45

**Search strategy**

First, from the preliminary search, we compared the final selected studies to studies listed in their references to reveal differences in indexing and limitations of the preliminary search. Aiming to cover the indexing and limitations, we then created the final search protocol with the aid of an information specialist by use of the population, intervention, comparison, outcome (PICO) format. Our study question in PICO format was: Do cholesterol and lipoprotein levels associate with risk for SAH? We checked reference lists of the selected studies (from the second search) and earlier reviews to identify additional relevant publications. We did the final search first time in October 2014 and then updated it in February, March, April, and June 2015. The latest update was done on 15th of December 2015.

Some data in reviewed studies was limited; although we requested it from authors of the largest studies, lack of response (with reasons discussed in Results and Discussion) meant that we could not conduct individual patient data analysis.

Variables collected: age, alcohol consumption, apo a levels, apo b levels, apo e levels, author, blood pressure levels, BMI levels, cancer, cholesterol levels, contraceptives, diabetes, diagnosis-verification, diet, exercise status, family history of stroke, fasting status, fasting time, HDL levels, heart disease, history of stroke, hypertension, hyperthyroidism, hypothyroidism, LDL levels, lipid lowering drugs, lipoproteins, liver disease, measurement methods, mean or median age at SAH, mean or median follow-up, mean or median time from measurement to SAH, menopausal status, number of fatal SAHs, number of females, number of males, number of non-fatal SAHs, number of participants, observed risk factors, outside-hospital deaths, SAH cases, SAH cases among men, SAH cases among women, sample size, sex, smoking levels, socioeconomic status, statistical methods, study year, time from measurement to SAH, type of control group.
