# Supplementary material for: Cholesterol as a Risk Factor for Subarachnoid Hemorrhage: A Systematic Review
Source: PLoS One. 2016 Apr 14;11(4):e0152568. doi: 10.1371/journal.pone.0152568 (PMC4831795; doi:10.1371/journal.pone.0152568)
Supplement: S1 Table — (DOCX) [file pone.0152568.s005.docx]

| **Supplemental Table 1** |  | |  |  |
| --- | --- | --- | --- | --- |
| **Cholesterol measurement protocols** | |  | | |
| **Study** | **Measurement*** | | **Device and method** | **Study design** † |
| **Finland** |  | |  |  |
| Korja 2013^3^ | SF | | 4 different methods‡ | P |
| Zhang 2012^6^ | SF | | 4 different methods‡ | P |
| Leppälä 1999^29^ | N | | TC:CHOD-PAP Boehringer-Mannheim HDL: dextran sulfate and magnesium chloride | P |
| Knekt 1991^1^ | SF | | Auto analysis Lieberman-Buchard | P |
| **Great Britain and Denmark** |  | |  | |
| Adamson 1994^25^ | N | | Mg-dextran precipitation | R |
| **Japan** |  | |  |  |
| Suzuki 2011^8^ | NR | | NR | P |
| Inagawa 2010^9^ | N | | NR | R |
| Cui 2007^27^ | N | | Automatic Analyzer Hitachi 7600-210, Hitachi Medical Corp., Hitachi, Japan | P |
| Inagawa 2005^4^ | N | | NR | R |
| Tokuda 2005^11^ | F | | "Automated biochemical system" | R |
| Ohkuma 2003^10^ | I | | I | R |
| **The Neatherlands** |  | |  | |
| Vlak 2013^12^ | I | | I | R |
| **Norway** |  | |  |  |
| Sandevei 2011^2^ | N | | Hitachi 911 auto analyzer CHOD-PAP Boeringer-Mannheim kit | P |
| **Portugal** |  | |  |  |
| Canhao 1994^26^ | N | | NR | R |
| **South Korea** |  | |  |  |
| Suh 2001^22^ | F | | NR | P |
| Park 1998^5^ | N | | NR | R |
| **Sweden** |  | |  |  |
| Gatchev 1993^7^ | N | | Lieberman Buchard in automatic multiple analyzer (AutoChemist) | P |
| **USA** |  | |  |  |
| Broderick 2003^23^ | I | | I | R |
| Neaton 1993^24^ | N | | Auto Analyzer II (Lieberman-Buchard) | P |
| Iso 1989^28^ | N | | Auto Analyzer II (Lieberman-Buchard) | P |
| Tirschwell 2004^30^ | NR | | NR | P |

*F=Fasting, SF=semi fasting, N=Non-fasting, I=interview, NR=not reported

†P=prospective R=retrospective

‡Two Finnish studies used following methods: TC: 1)1972-77 Lieberman-Buchard 2) 1982- CHOD-PAP Boeringer- Mannheim kit HDL 1)1982-1997 dextran- sulfate and Mg OlliC 2) Direct method (Thermo Fisher Scientific), Optima clinical chemistry analyzer
